# Physical activity and sedentary time are related to clinically relevant health outcomes among adults with obstructive lung disease

**DOI:** 10.1186/s12890-018-0659-8

**Published:** 2018-06-07

**Authors:** Shilpa Dogra, Joshua Good, Matthew P. Buman, Paul A. Gardiner, Jennifer L. Copeland, Michael K. Stickland

**Affiliations:** 1 Faculty of Health Sciences (Kinesiology), University of Ontario Institute of Technology, 2000 Simcoe St N, Oshawa, ON L1H-7K4 Canada; 2 College of Health Solutions, Arizona State University, 550 N 3rd Street, Phoenix, AZ 85004 USA; 3 Faculty of Medicine, The University of Queensland, Level 2, Building 33, Princess Alexandra Hospital, Woolloongabba, QLD 4102 Australia; 4 Department of Kinesiology and Physical Education, University of Lethbridge, 4401 University Drive, Lethbridge, AB T1K 3M4 Canada; 5 Faculty of Medicine and Dentistry, University of Alberta, and G.F. Macdonald Centre for Lung Health, 3-135 Clinical Sciences Building, 11304 - 83 Avenue, Edmonton, Alberta T6G 2J3 Canada

**Keywords:** Asthma, COPD, Physical activity, Hospitalization

## Abstract

**Background:**

The purpose of the current study was to determine the association between sedentary time and physical activity with clinically relevant health outcomes among adults with impaired spirometry and those with or without self-reported obstructive lung disease (asthma or COPD).

**Methods:**

Data from participants of the Canadian Longitudinal Study on Aging were used for analysis (*n* = 4156). Lung function was assessed using spirometry. Adults were said to have impaired spirometry if their Forced Expiratory Volume in 1 s was <5th percentile lower limit of normal (LLN). A modified version of the Physical Activity Scale for the Elderly was used to assess sitting time and physical activity levels. Healthcare use and quality of life outcomes were assessed using self report.

**Results:**

Among those with asthma, participating in strengthening activities was associated with lower odds of reporting poor perceived health (OR = 0.65, CI: 0.53, 0.79), poor perceived mental-health (OR = 0.73, CI: 0.60, 0.88), unhealthy aging (OR = 0.68, CI: 0.56, 0.83), and reporting an emergency department visit in the past 12 months (OR = 0.76, CI: 0.60, 0.95). Among those with COPD, those who reported highest weekly sedentary time had higher odds of reporting poor perceived health (OR = 2.70, CI: 1.72, 4.24), poor perceived mental-health (OR = 1.99, CI: 1.29, 3.06), and unhealthy aging (OR = 3.04, CI: 1.96, 4.72). Among those below the LLN, sitting time (OR = 2.57, CI: 1.40, 4.72) and moderate intensity physical activity (OR = 0.23, CI: 0.09, 0.63) were associated with overnight hospital stays.

**Conclusions:**

Higher physical activity levels and lower sedentary time may be associated with lower healthcare use and better quality of life. This research may have implications related to the use of physical activity for improving health outcomes and quality of life among adults with obstructive lung disease or impaired spirometry.

**Electronic supplementary material:**

The online version of this article (10.1186/s12890-018-0659-8) contains supplementary material, which is available to authorized users.

## Background

Chronic obstructive lung diseases such as asthma and chronic obstructive pulmonary disease (COPD) are estimated to affect 4.3 and 4.7% of the global population, respectively [1, 2], with the prevalence as high as 20–25% in some countries [1, 3]. Obstructive lung diseases are typically associated with suboptimal quality of life, and an increased burden on the healthcare system [4, 5]. Importantly, adults with chronic lung diseases who are physically active have better health outcomes, and lower health care use than their inactive peers [6–8]. Among those with COPD, systematic reviews of the literature have shown that physical inactivity is associated with worse lung function [9], lower health-related quality of life, and greater dyspnea [10]. Similarly, while regular exercise is not associated with improved lung function among adults with asthma, it leads to significant improvements in health related quality of life [11].

Evidence suggests that individuals with obstructive lung disease are often misdiagnosed using previously established fixed ratio lung function cut-points [12]. It has been suggested that clinicians use the lower limit of normal (LLN), that is, spirometry values below the 5th percentile, instead [12]. Not surprisingly, the risk of hospitalization is higher among those with COPD who are below the LLN compared to those above the LLN [13]. However, no evidence is available on the association between physical activity and health outcomes among those below the LLN, regardless of whether they have a diagnosed lung disease.

There is also a dearth of research on the association between sedentary time and health outcomes among those with obstructive lung disease. Sedentary behavior is any activity performed in a seated or reclined position requiring low energy expenditure while awake [14], and a growing body of literature suggests an association between time spent in sedentary activities and health [15], particularly among older adults [16]. Among those with COPD and asthma, sedentary time may be associated with dyspnea, higher health care use, worse disease management, and all-cause mortality [17–20]. Recent evidence suggests that both physical activity and sedentary time are associated with lung function among healthy adults (Dogra S, Good J, Buman MP, Gardiner P, Stickland MK, Copeland J. Movement behaviours are associated with lung function in middle-aged and older adults: a cross-sectional analysis of the Canadian longitudinal study on aging, unpublished). However, the association between sedentary time and clinically relevant health outcomes among those with existing obstructive lung diseases or among those with impaired spirometry is not known.

Physical activity levels among those with obstructive lung disease remain suboptimal [21], and sedentary time is likely high due to dyspnea and deconditioning. Both sedentary time and physical activity may be modifiable determinants of clinically relevant health outcomes among those with obstructive lung disease or those with impaired spirometry, and could be used clinically to understand symptom management. However, large database analysis is needed to establish correlations before clinical investigation can be undertaken. Thus, the purpose of the present analysis was to determine the association between sedentary time as well as different modes and intensities of physical activity with clinically relevant outcomes of lung function, healthcare use, and quality of life, among middle-aged and older adults with self-reported obstructive lung disease (i.e. COPD, asthma). We also examined these associations separately among those who had impaired spirometry as per the LLN, regardless of whether they had a diagnosed lung disease.

## Methods

### Data source and participants

The Canadian Longitudinal Study on Aging (CLSA) is a nationally representative, stratified, random sample of 51,338 Canadian women and men aged 45 to 85 years (at baseline). The purpose of this survey is to collect data on the health and quality of life of Canadians to better understand the processes and dimensions of aging. The study contains two samples: the CLSA Comprehensive, and the CLSA Tracking. Data from participants in the first sample were collected through questionnaires, physical examinations and biological samples. These participants live within a 25-50 km radius of one of the 11 data collection sites across Canada (Vancouver/Surrey (two sites), Victoria, Calgary, Winnipeg, Hamilton, Ottawa, Montreal, Sherbrooke, Halifax, and St. John’s). This sample contains approximately 30,000 participants, recruited between 2012 and 2015, and was used for the current study.

Inclusion in the CLSA was limited to those who were able to read and speak either French or English. Residents in the three territories and some remote regions, persons living on federal First Nations reserves and other First Nations settlements in the provinces, and full-time members of the Canadian Armed Forces were excluded. Individuals living in long-term care institutions (i.e., those providing 24-h nursing care) were excluded at baseline; however, those living in households and transitional housing arrangements (e.g., seniors’ residences, in which only minimal care is provided) were included. Finally, those with a cognitive impairment at the time of recruitment were excluded.

The protocol of the CLSA has been reviewed and approved by 13 research ethics boards across Canada. Changes to the CLSA protocol are reviewed annually. Written consent is obtained from all participants. The University of Ontario Institute of Technology Research Ethics Board approved secondary analysis of the CLSA dataset (REB #1367).

### Measures

#### Outcome variables

##### Forced expiratory volume in 1 s (FEV_1_)

Spirometry was conducted using the TruFlow Easy-On Spirometer. Only those with major contraindications did not perform the test [22]. Maximal inspiratory and expiratory maneuvers were performed to obtain FEV_1_ and forced vital capacity (FVC). Only participants who performed at least three acceptable efforts, with their best two FVC and FEV_1_ within 150 ml, were included. The best FEV_1_ and FVC were used for analysis.

##### Healthcare use

Participants were asked “Have you been seen in an Emergency Department during the past 12 months?” and “Were you a patient in a hospital overnight during the past 12 months?”. Response options were yes or no.

##### Quality of life

Participants were asked “In general, would you say your health is excellent, very good, good, fair, or poor?”, “In general, would you say your mental health is excellent, very good, good, fair, or poor?”, and “In terms of your own healthy aging, would you say it is excellent, very good, good, fair, or poor?”. Each variable was re-categorized in to “Good” (excellent, or very good,) and “Poor” (good, fair, or poor) based on the distribution of the sample.

#### Lung disease categories

Participants were asked whether a doctor had ever told them that they have asthma, and whether a doctor had ever told them that they have emphysema, chronic bronchitis, chronic obstructive pulmonary disease (COPD), or chronic changes to their lungs due to smoking. Participants who responded “yes” to either question were considered to have an obstructive lung disease, regardless of spirometry data. Thus, asthma and COPD were self-reported.

Participants with a FEV_1_ > 10 Litres were excluded. Predicted FEV_1_ (FEV_1%pred_) and LLN were calculated based on age, height, and sex using formulas developed on the Canadian population [23]. The LLN for each participant was calculated using the formula:

$$ \mathrm{LLN}=\mathrm{predicted}\ \mathrm{value}-\left(1.645\times \mathrm{Standard}\ \mathrm{Error}\ \mathrm{of}\ \mathrm{the}\ \mathrm{Estimate}\right) $$

Individuals with an FEV_1_ < 5th percentile LLN were considered to have impaired spirometry, regardless of whether they reported a diagnosed obstructive lung disease*.*

#### Exposure variables

##### Physical activity and sitting time

A modified version of the Physical Activity Scale for Elderly (PASE) was used to collect information on sitting time and physical activity. The PASE is a valid and reliable tool for assessing physical activity and sitting time among older adults. It has been shown to have good test-retest reliability over a 3 to 7-week interval (0.75, 95% CI = 0.69–0.80). Construct validity has also been established [24].

With regard to sitting time, participants were asked “Over the past 7 days, how often did you participate in sitting activities such as reading, watching TV, computer activities or doing handicrafts?” and “On average, how many hours per day did you engage in these sitting activities?”. The frequency of individual sitting activities was recorded in categories of never, seldom (1 to 2 days), sometimes (3 to 4 days), or often (5 to 7 days) for frequency, and the duration of individual sitting activities was recorded in categories of < 30 min, 30 min to < 1 h, 1 h to < 2 h, 2 h to < 4 h, or 4 h or more. The midpoint of each frequency and duration category (except for the 4 h or more hours category, which was coded as 4 h), was used to estimate weekly total sitting time in hours per week.

The PASE also asks a series of questions pertaining to physical activity over the past 7 days. Specifically, participants were asked how often they took a walk outside, engaged in light sports or recreational activities, engaged in moderate sports or recreational activities, engaged in strenuous sports or recreational activities, and engaged in exercises specifically to increase muscle strength and endurance. The frequency and duration for each activity was recorded in the same way as for sitting time; the same midpoints were used to calculate hours per week spent in each type/intensity of activity.

Weekly physical activity and sitting time variables were categorized for logistic regressions. For sitting time (0 to 14 h, 14.1 to 18 h, and 18.1 to 24 h) and walking (0 to 2.25 h, 2.26 to 4.5 h, 4.6 to 24 h), there was enough variability to categorize the variables based on tertiles. For light intensity PA (0 h, > 0 h), moderate intensity physical activity (0 h, > 0 h), strenuous intensity PA (0 h, > 0 h), and muscle strengthening activity (0 h, > 0 h), variables were dichotomized due to lack of variability.

#### Covariates

##### Smoking status

Pack years were calculated using eight variables from the CLSA. Participants who responded negatively to “Have you smoked at least 100 cigarettes in your life? (about 4 - 5 packs)” were categorized as “Never Smoked”. Participants who were current smokers were asked “For how many total years have you smoked daily?” and “During the total years that you have smoked daily, about how many cigarettes per day have you usually smoked? (If your smoking pattern has changed over the years, make your best guess of the average number of cigarettes you have smoked per day.)” The number of cigarettes smoked per day was recorded in categories of 1–5, 6–10, 11–15, 16–20, 21–25, and 26+ cigarettes. The midpoint of each of category was used to determine the number of cigarettes smoked per day with the exception of 26+ cigarettes in which case an exact number was recorded. Similar questions were asked to former daily smokers.

Pack years was calculated as: [(number of cigarettes smoked per day/20 cigarettes per pack) x number of years smoked]. Pack years was then categorized into Never Smoked, < 10 pack years, and 10 or more pack years. Participants who were never daily smokers but had smoked more than 100 lifetime cigarettes were included in the < 10 pack years category.

##### Others

Participants were asked to report their age and sex, and provided information on several additional relevant covariates. For *sleep*, participants were asked “During the past month, on average, how many hours of actual sleep did you get at night?”. This was categorized into < 6 h, 6–8 h, and > 8 h according to previous research on the association between sleep and health [25]. For *retirement status*, participants were asked “At this time, do you consider yourself to be completely retired, partly retired or not retired”. Those who responded partly retired were merged with the not retired group due to sample size. For *education levels*, participants were asked four questions pertaining to their highest level of education. These responses were combined to categorize the sample as: Less than secondary school graduation, secondary school graduation, no post-secondary education, some post-secondary education, or post-secondary degree/diploma. Height and weight were measured by trained professionals, and used to calculate *body mass index* (kg/m^2^).

For the present analysis, only those who reported an obstructive lung disease (defined above, *n* = 5094) or those with impaired spirometry (*n* = 1747) were included for analysis. Of note, participants may have been in multiple groups, that is, those with asthma may also have reported COPD, or had an FEV_1_ below the LLN. Those who reported a history of lung cancer (*n* = 103) were not included, and only those with complete data for spirometry (*n* = 4493), physical activity and sitting time, quality of life, healthcare use (*n* = 4212), and all covariates (*n* = 4156) were included. In this select sample, 1939 had asthma only, 432 had COPD only, and 1021 were below LLN; 764 were in multiple groups. Of those with COPD, only 224 were below the LLN. Individuals who responded positively to “Have you taken any long acting inhalers in the last 12 hours?” and/or “Have you taken any short acting inhalers in the last 6 hours?” were excluded (*n* = 713) from analyses where spirometry was the main outcome.

### Statistical analysis

Means and frequencies were used to describe the sample. Crude beta coefficients for the associations of FEV_1%pred_ and FEV_1_/FVC with sitting time, walking, light physical activity, moderate physical activity, strenuous physical activity, and muscle strengthening activity were assessed using linear regression models. Hierarchical models were used to generate adjusted associations for FEV_1%pred_. Specifically, block 1 contained all of the covariates (age, sex, sleep, retirement status, education level, and body mass index) while block 2 included each of the sedentary and physical activity variables. Models (containing both blocks) were run separately for those with asthma, COPD, and those who demonstrated impaired spirometry (FEV_1_ below LLN).

Crude and adjusted odds ratios were calculated for quality of life (perceived general health, perceived mental health, and healthy aging) and healthcare use outcomes (emergency department visit and overnight hospitalization) for sitting time, walking, light physical activity, moderate physical activity, strenuous physical activity, and muscle strengthening activity using logistic regression models. Adjusted models included age, sex, sleep, retirement status, education level, body mass index, and FEV_1%pred_. Models were run separately for those with asthma, COPD, and those with impaired spirometry (FEV_1_ below LLN).

All analyses were performed using SPSS v.24. To ensure national representation and to compensate for under-represented groups, sampling weights were applied to regression models. Significance was set at *p* < 0.05. Additional details on sampling, methods and weighting on the CLSA can be found in the protocol document [26, 27].

## Results

The overall sample was 61.6 ± 9.9 years of age, with 45.5% being male. Per week, participants were averaging 18.3 ± 6.1 h of sitting time, 4.2 ± 4.6 h of walking, 0.8 ± 2.5 h of light intensity physical activity, 0.7 ± 2.4 h of moderate intensity physical activity, 1.3 ± 2.9 h of strenuous intensity physical activity, and 0.7 ± 1.7 h of strengthening activity. The sample had an average FEV_1%pred_ of 84.4 ± 19.2%. Additional sample characteristics can be found by lung condition in Table 1.

**Table 1:** Sample Characteristics of Adults with Asthma, COPD, and those below the LLN

Characteristics		Asthma (*n* = 2569)	COPD (*n* = 877)	Below LLN for FEV_1_ (*n* = 1545)
Age (years)		60.9 ± 9.7	65.0 ± 9.9	61.7 ± 9.9
BMI (kg/m^2^)		29.0 ± 6.1	29.2 ± 6.3	29.6 ± 6.6
Height (cm)		167.0 ± 9.6	166.3 ± 9.5	170.8 ± 9.7
FEV_1_ (L)		2.5 ± 0.7	2.2 ± 0.8	2.0 ± 0.6
FEV_1_% predicted		91.2 ± 17.3	85.1 ± 20.4	65.4 ± 9.7
FVC (L)		3.4 ± 0.9	3.1 ± 0.9	2.9 ± 0.8
FVC % predicted		90.6 ± 14.6	85.9 ± 16.1	70.6 ± 10.8
FEV_1_/FVC		0.75 ± 0.07	0.72 ± 0.09	0.69 ± 0.09
FEV_1_/FVC % predicted		99.9 ± 9.4	97.3 ± 12.0	92.9 ± 12.0
Education (% of sample)	Less than secondary school graduation	4.0%	9.2%	6.2%
	Secondary school graduation, no post-secondary education	7.3%	10.3%	9.6%
	Some post-secondary education	6.8%	9.9%	8.3%
	Post-secondary degree/diploma	81.9%	70.6%	75.9%
Retirement Status (% of sample)	Retired	38.1%	54.0%	41.1%
	Not or partly retired	61.9%	46.0%	58.9%
Activity Levels (hours/week)	In sitting activities	18.2 ± 6.1	19.1 ± 5.9	18.5 ± 6.1
	Walking	4.3 ± 4.7	3.9 ± 4.3	4.1 ± 4.7
	Light activities	0.8 ± 2.5	0.9 ± 2.6	0.8 ± 2.6
	Moderate sports or recreational activities	0.7 ± 2.4	0.7 ± 2.6	0.6 ± 2.3
	Strenuous sports or recreational activities	1.5 ± 3.0	0.8 ± 2.2	1.1 ± 2.7
	Increase muscle strength and endurance	0.7 ± 1.7	0.7 ± 1.9	0.6 ± 1.5
Sleep (% of sample)	Less than 6 h	14.4%	19.8%	14.4%
	6 to 8 h	80.1%	71.2%	80.8%
	More than 8 h	5.4%	9.0%	4.8%
Perceived health (% of sample)	Good (excellent and very good	56.3%	42.9%	48.3%
	Poor (good, fair, and poor)	43.7%	57.1%	51.7%
Self-rated healthy aging (% of sample)	Good (excellent and very good	59.0%	46.2%	49.6%
	Poor (good, fair, and poor)	41.0%	53.8%	50.4%
Self-rated mental health (% of sample)	Good (excellent and very good	66.6%	57.8%	66.0%
	Poor (good, fair, and poor)	33.4%	42.2%	34.0%
Emergency visit in last 12 months (% Yes)		21.1%	26.7%	20.5%
Overnight hospitalization in last 12 months (% Yes)		8.8%	12.5%	9.9%

**Fig. 1:**
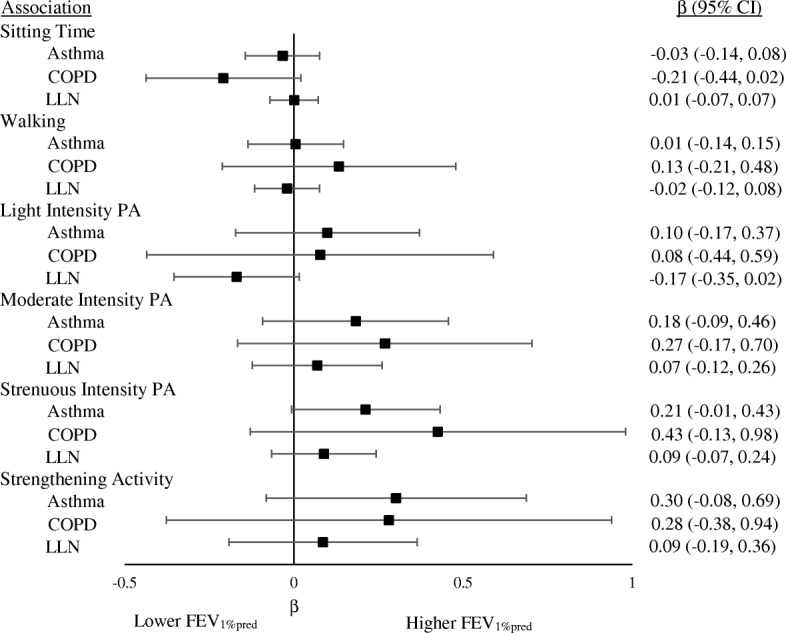
Adjusted associations of FEV_1%pred_ with Sitting Time and Physical Activity for adults with Asthma, COPD, and those below the LLN. Note: Covariates included in adjusted models were age, sex, sleep, retirement status, education level, and body mass index. PA: Physical Activity; *p < 0.05, **p < 0.01, ***p < 0.001

In crude models assessing the association with FEV_1%pred_, participating in strenuous intensity physical activity was associated with better FEV_1%pred_ among those with asthma (β: 0.31, CI: 1.00–0.52), COPD (β: 0.78, CI: 0.21–1.35), and those below the LLN (β: 0.27, CI: 0.11–0.42) (Additional file 1: Figure S1). There were no significant associations once models were adjusted for covariates (age, sex, sleep, retirement status, education level, and body mass index, Fig. 1). The association between sitting time and FEV_1_/FVC was significant among those with COPD (β: -0.18, CI:-0.28, − 0.08) such that a higher FEV_1_/FVC was associated with lower sitting time. A significant association was also noted between FEV_1_/FVC with strenuous intensity physical activity, such that those with COPD participating in strenuous intensity physical activity had a higher FEV_1_/FVC. Finally, there was an inverse association with light intensity physical activity such that those below the LLN participating in light intensity physical activity had lower FEV_1_/FVC. No other associations were significant in crude models (Additional file 2: Figure S2).

The associations for healthcare use outcomes are presented in Table 2. In crude models, sedentary time, walking, moderate intensity, and strenuous intensity physical activity were associated with overnight hospital stays in both adults with asthma and those below the LLN. In models adjusted for covariates (age, sex, sleep, retirement status, education level, body mass index, and FEV_1%pred_), participating in light intensity activity (OR: 0.61, CI: 0.40–0.92) was associated with lower odds of reporting an overnight hospital stay among those with asthma compared to those who were not physically active at light intensities. Among those below the LLN, sedentary time appeared to be important for overnight hospital stays such that those who reported 14.1–18 h/week (OR: 2.03, CI: 1.09–3.78) and those who reported 18.1–24 h per week (OR: 2.57, CI: 1.40–4.72) were approximately 2 times more likely to report an overnight hospital stay than those who reported < 14 h per week of sedentary time.

**Table 2:** Associations of Healthcare Use with Sitting Time and Physical Activity among adults with Asthma, COPD, and those below the LLN

		Asthma		COPD		Below LLN for FEV_1_	
		Overnight hospital visit	Emergency department visit	Overnight hospital visit	Emergency department visit	Overnight hospital visit	Emergency department visit
Activity (referent category)	Category	OR (95% CI)	OR (95% CI)	OR (95% CI)	OR (95% CI)	OR (95% CI)	OR (95% CI)
a) Crude associations							
Sitting Time (14 h/ week or less)	14.1 to 18 h/ week	1.10 (0.74–1.63)	1.16 (0.90–1.49)	1.10 (0.55–2.20)	1.42 (0.89–2.26)	2.40** (1.30–4.43)	1.45* (1.02–2.05)
	18.1 to 24 h/ week	1.63* (1.12–2.38)	1.32* (1.03–1.70)	1.70 (0.89–3.25)	1.44 (0.91–2.27)	3.48*** (1.92–6.29)	1.39 (0.99–1.97)
Walking (2.25 h/week or less)	2.26 to 4.5 h/ week	0.62** (0.45–0.86)	0.78* (0.62–0.96)	0.67 (0.38–1.18)	0.93 (0.64–1.37)	0.65* (0.43–0.99)	0.81 (0.60–1.10)
	4.6 to 24 h/ week	0.58** (0.40–0.83)	0.86 (0.68–1.08)	1.04 (0.61–1.75)	1.15 (0.78–1.69)	0.55* (0.35–0.87)	0.76 (0.56–1.04)
Light Intensity PA (No time reported)	Greater than 0 h/ week	0.58** (0.39–0.86)	0.90 (0.71–1.14)	1.07 (0.63–1.83)	1.75** (1.21–2.52)	0.77 (0.47–1.25)	1.15 (0.84–1.58)
Moderate Intensity PA (No time reported)	Greater than 0 h/ week	0.49** (0.29–0.83)	0.76 (0.57–1.03)	0.61 (0.27–1.36)	0.76 (0.46–1.26)	0.22** (0.08–0.59)	0.82 (0.55–1.24)
Strenuous PA (No time reported)	Greater than 0 h/ week	0.57*** (0.41–0.78)	0.78* (0.64–0.95)	0.50* (0.27–0.92)	1.01 (0.70–1.45)	0.53** (0.34–0.81)	0.79 (0.60–1.04)
Strengthening Activity (No time reported)	Greater than 0 h/ week	0.87 (0.63–1.19)	0.70** (0.56–0.87)	0.94 (0.56–1.59)	1.22 (0.85–1.75)	0.83 (0.55–1.25)	1.15 (0.87–1.52)
b) Adjusted associations							
Sitting Time (14 h/ week or less)	14.1 to 18 h/ week	0.92 (0.61–1.38)	1.07 (0.82–1.38)	1.08 (0.52–2.22)	1.41 (0.87–2.29)	2.03* (1.09–3.78)	1.38 (0.97–1.97)
	18.1 to 24 h/ week	1.04 (0.69–1.55)	1.11 (0.85–1.44)	1.25 (0.63–2.51)	1.39 (0.86–2.26)	2.57** (1.40–4.72)	1.30 (0.91–1.87)
Walking (2.25 h/week or less)	2.26 to 4.5 h/ week	0.75 (0.53–1.05)	0.86 (0.68–1.08)	0.80 (0.43–1.47)	0.97 (0.65–1.46)	0.74 (0.47–1.14)	0.84 (0.62–1.14)
	4.6 to 24 h/ week	0.69 (0.47–1.00)	0.93 (0.74–1.18)	1.31 (0.75–2.30)	1.20 (0.80–1.81)	0.70 (0.43–1.12)	0.82 (0.59–1.12)
Light Intensity PA (No time reported)	Greater than 0 h/ week	0.61* (0.40–0.92)	0.95 (0.75–1.20)	1.28 (0.73–2.27)	1.99** (1.35–2.93)	0.95 (0.57–1.56)	1.25 (0.90–1.73)
Moderate Intensity PA (No time reported)	Greater than 0 h/ week	0.58 (0.34–1.00)	0.85 (0.63–1.14)	0.74 (0.32–1.73)	0.75 (0.43–1.29)	0.23** (0.09–0.63)	0.85 (0.56–1.29)
Strenuous PA (No time reported)	Greater than 0 h/ week	0.80 (0.56–1.13)	1.01 (0.81–1.25)	0.61 (0.32–1.16)	0.99 (0.67–1.48)	0.69 (0.44–1.11)	0.86 (0.63–1.17)
Strengthening Activity (No time reported)	Greater than 0 h/ week	1.13 (0.80–1.58)	0.76* (0.60–0.95)	1.20 (0.68–2.11)	1.37 (0.93–2.01)	1.08 (0.70–1.69)	1.35* (1.00–1.82)

A higher OR indicates a higher odds of having an overnight hospitalization or emergency room visit in the last 12 months relative to the referent category (as indicated in title). Covariates included in adjusted models were age, sex, sleep, retirement status, education level, body mass index, and FEV_1%pred_. PA: Physical Activity; *p < 0.05, **p < 0.01, ***p < 0.001

**Fig. 2:**
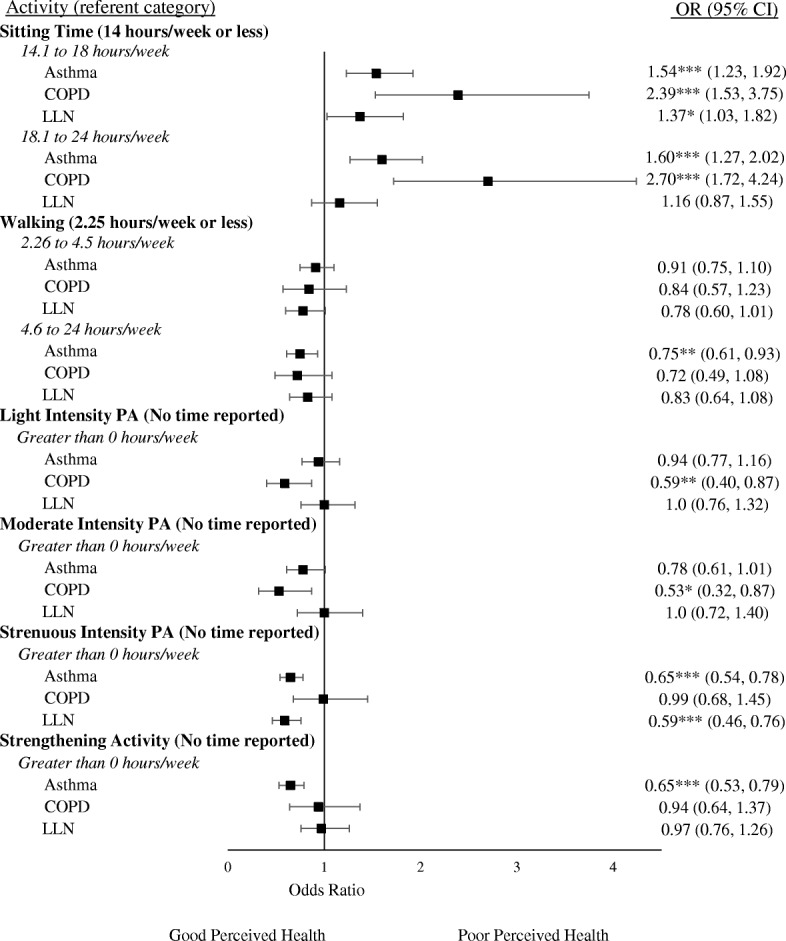
Adjusted associations of Self-perceived health with Sitting Time and Physical Activity among adults with Asthma, COPD, and those below the LLN. Note: A higher OR indicates a higher odds of having “Poor” self-perceived health relative to the referent category. Covariates included in adjusted models were age, sex, sleep, retirement status, education level, body mass index, and FEV_1%pred_. PA: Physical Activity; *p < 0.05, **p < 0.01, ***p < 0.001

**Fig. 3:**
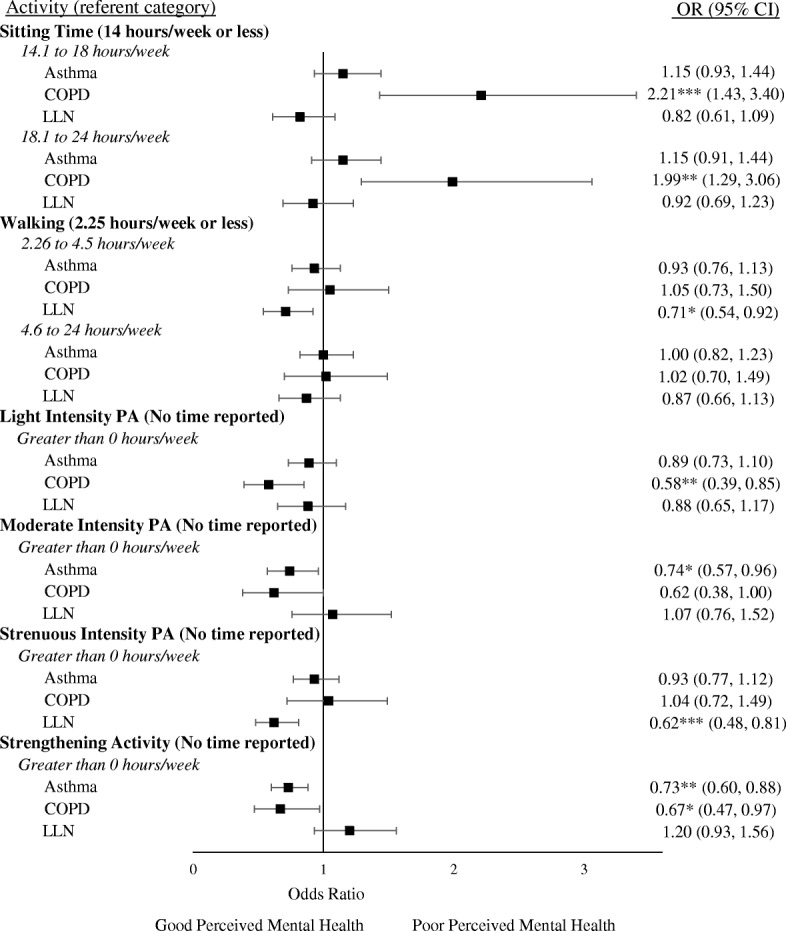
Adjusted associations of Self-perceived mental health with Sitting Time and Physical Activity among adults with Asthma, COPD, and those below the LLN. Note: A higher OR indicates a higher odds of having “Poor” self-perceived mental health relative to the referent category. Covariates included in adjusted models were age, sex, sleep, retirement status, education level, body mass index, and FEV_1%pred_. PA: Physical Activity; *p < 0.05, **p < 0.01, ***p < 0.001

**Fig. 4:**
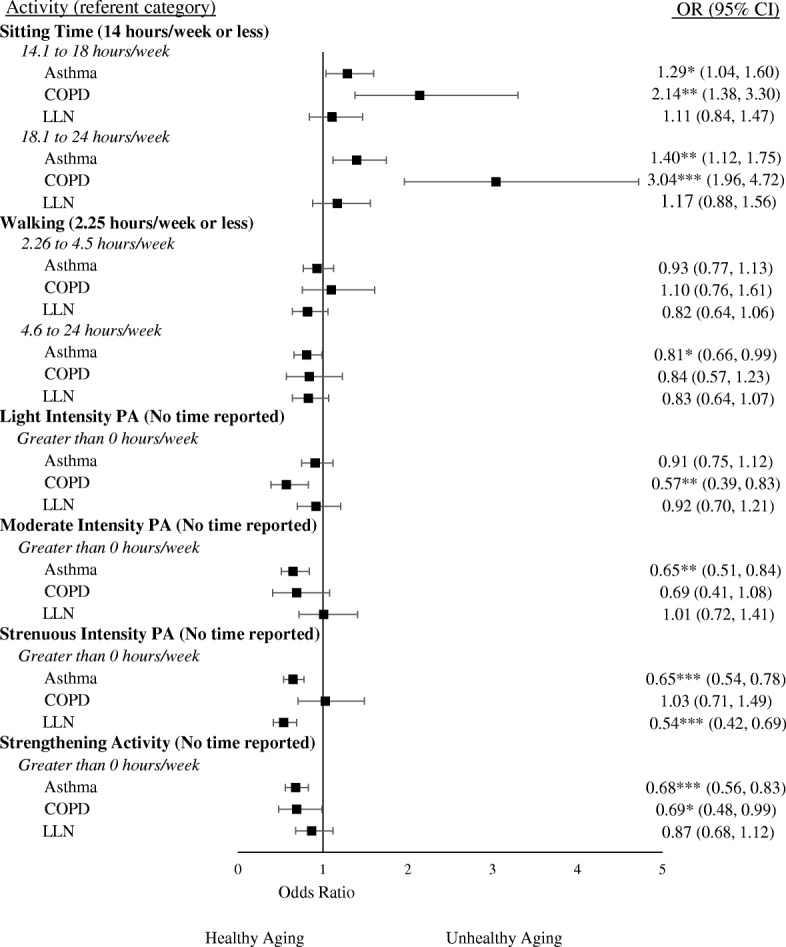
Adjusted associations of Self-perceived healthy aging with Sitting Time and Physical Activity among adults with Asthma, COPD, and those below the LLN. Note: A higher OR indicates a higher odds of having “Poor” self-perceived healthy aging relative to the referent category. Covariates included in adjusted models were age, sex, sleep, retirement status, education level, body mass index, and FEV_1%pred_. PA: Physical Activity; *p < 0.05, **p < 0.01, ***p < 0.001

Associations for poor perceived health, poor perceived mental health, and unhealthy aging are presented in Figs. 2, 3 and 4 (crude associations are presented in Additional file 3: Figure S3, Additional file 4: Figure S4, Additional file 5: Figure S5). Among those with asthma, participating in strengthening activities was associated with lower odds of reporting poor perceived health (OR: 0.65, CI: 0.53–0.79), poor perceived mental-health (OR: 0.73, CI: 0.60–0.88), and unhealthy aging (OR: 0.68, CI: 0.56–0.83) compared to those who did not participate in strengthening activity in fully adjusted models. Among those with COPD, those who reported higher weekly sedentary time had higher odds of reporting poor perceived health, poor perceived mental-health, and unhealthy aging in fully adjusted models. Finally, among those below the LLN, participating in strenuous intensity physical activity was associated with lower odds of reporting poor perceived health (OR: 0.59, CI: 0.46–0.76), perceived mental-health (OR: 0.62, CI: 0.48–0.81), and unhealthy aging (OR: 0.54, CI: 0.42–0.69) compared to those who did not participate in strenuous physical activity in fully adjusted models.

## Discussion

These data are the first, to our knowledge, to assess the associations of sitting time, strenuous intensity physical activity, and strengthening activity among adults with obstructive lung disease, and the first to conduct such analyses among those who fall below the LLN for FEV_1_. Our primary finding is that physical activity and sedentary time *may be* important risk factors for hospitalization. Specifically, light intensity physical activity was associated with lower odds of reporting an overnight hospital stay, and strengthening activity was associated with lower odds of an emergency department visit among adults with asthma. Further, among those with a FEV_1_ below the LLN, engaging in less than 14 h of sedentary time or participating in moderate intensity physical activity were associated with lower odds of reporting an overnight hospital stay. In addition to hospitalization, physical activity and sedentary time were consistently associated with quality of life measures amongst the three groups. These findings provide insight into the importance of physical activity and sedentary time for disease management among individuals with obstructive lung disease or among those with impaired spirometry.

### Asthma

The association between physical activity and clinically relevant health outcomes among adults with asthma is not surprising [6, 19], however, associations with sedentary time and strengthening activity are poorly studied. To date, most of the available literature in the area of asthma and sedentary time is from young samples [28]. In a Canadian study of Indigenous adults, it was found that healthcare use was higher among those who watched more than 10 h of television per week after adjustment for physical activity [19]. Little additional data are available in adults. Similarly, for the association between strengthening activity and asthma outcomes, evidence from RCTs in obese adults with asthma indicate that concurrent strengthening and endurance training improve a variety of asthma specific outcomes [29], however the independent effect of strength training is not clear. Strength training may be a particularly important activity to assess among adults with asthma as it has a low ventilatory requirement and thus less likely to induce bronchoconstriction. Given that adults with asthma tend to be less active than their peers, reducing sedentary time or engaging in strengthening activities may be an effective method for initiating an active lifestyle. The present study highlights several opportunities for future research among adults with asthma.

### Chronic obstructive pulmonary disease

Associations between physical activity and a variety of quality of life and healthcare use measures have been previously reported among adults with COPD [30–33]. The novelty of the present study is the assessment of different intensities of physical activity, as well the inclusion of strengthening activity and sedentary time. Previous work has found that light intensity physical activity, but not high intensity physical activity, is important for hospitalization avoidance among those with COPD [33]. Surprisingly, the current study found that engaging in light intensity physical activity was associated with *increased* risk of emergency department visits in COPD patients, whereas no association was found between moderate or strenuous physical activity and emergency department visits. Previous work that found that light intensity physical activity was associated with hospitalization avoidance used an activity monitor (Sensewear Armband) to record physical activity, whereas the CLSA uses a self-reported tool. Self-reported physical activity may lack precision, and additional follow-up studies using physical activity monitors are needed to better understand how different intensities of physical activity relate to healthcare utilization in COPD. Furthermore, it is possible that those with severe COPD experienced exacerbations or severe dyspnea due to engagement in light intensity physical activity, thus increasing their odds of emergency medical needs. Of note, the examples provided for light intensity physical activity in the questionnaire (eg: bowling, golf with a cart) may be of higher relative intensity for individuals with severe COPD. Future research is needed to better understand the effects of disease severity and comorbidity on healthcare use outcomes.

While we did not observe an association between strenuous activity and hospitalizations, it should be noted that strenuous intensity physical activity is beneficial for dyspnea and ventilatory parameters [34], and leads to significant improvements in exercise capacity [35]. Higher exercise capacity is also associated with better health outcomes in COPD [36]. Therefore, future research should further investigate the potential role of strenuous intensity physical activity for COPD management.

The relationship between exercise capacity and health outcomes may further explain why strength training was important among those with COPD. Some research has shown that when compared to endurance training, strength training has the same effect on exercise capacity in COPD [37]; this may be explained by the marked skeletal muscle dysfunction in COPD [38]. Skeletal muscle dysfunction contributes to dyspnea, which may explain why sitting time was associated with quality of life measures and healthcare use measures in our sample. Specifically, poor lung function or disease management may lead to an increase in sedentary behaviours to avoid dyspnea. Although it has been documented that those with COPD have lower physical activity levels and higher sedentary time [39], no research to date has assessed the association of sedentary time with clinically relevant health outcomes in COPD.

### Impaired spirometry

Only one other study, to our knowledge, has looked at the association between sedentary time and health outcomes in those with spirometry values below the LLN. Vas Fragoso and colleagues [40] found that among community dwelling older adults, respiratory impairment (defined using FEV_1_ < LLN) was associated with sedentary time and mobility impairment. Although we did not assess mobility impairment, our finding that strenuous intensity physical activity was important for perceived health and healthy aging suggests that perhaps exercise capacity or mobility are critical indicators of the impact that declining lung function has on functional ability and quality of life. In addition, perceived health has been associated with chronic conditions such as depression, anxiety, and mortality [41–44]. Therefore strenuous physical activity may be important for potential comorbidities among individuals with poor lung function. This study is the first to our knowledge to report on sedentary time and physical activity among adults below the LLN, and indicates that both sitting time and strenuous intensity physical activity may be indicative of symptom management, and could be a valuable clinical tool.

Strengths of this study include the large sample of adults with asthma and COPD, the separate analysis of those with impaired spirometry (i.e. FEV_1_ below the LLN), and use of a validated questionnaire for sitting time and physical activity. Nevertheless, device- measured physical activity and sitting time would provide more valid data, as well as the opportunity to analyze differences between prolonged and interrupted sitting time. Similarly, data on cardiorespiratory and musculoskeletal fitness would have allowed for better understanding of the impact that physical activity and sedentary time have on health outcomes, as both physical activity and sedentary time are known to impact fitness levels, and fitness is likely a stronger predictor than the behaviours assessed.

Another limitation is the self-reported asthma, COPD, and healthcare use variables. It is possible that participants did not accurately recall hospital stays or emergency department visits; however, given that these are prominent events, and that response options were yes or no, the risk of misclassification is low. Furthermore, we did not have data on walk-in clinic use, which may be important among those with poorly controlled disease. It should also be noted that healthcare use could have been related to other comorbidities. While we did remove those with lung cancer, we did not adjust for additional chronic conditions. Thus, care should be taken when interpreting the findings, as individuals with COPD tend to have more comorbidities than individuals with asthma. Another limitation is that the FVC appears to be underestimated as the mean FVC_%predicted_ in the overall CLSA cohort was 93% (85.0 ± 16.3 in the selected sample for this study). Although the FVC data were reproducible, the reduced FVC suggests that participants may not have exhaled for the maximal possible duration, thereby overestimating the FEV_1_/FVC and under reporting the degree of airflow obstruction.

Finally, it is important to note that data from the CLSA are cross-sectional, thus, reverse-causality cannot be ruled out at this time. In the next 5 years, the CLSA will provide its first round of longitudinal data where these associations can be further analyzed. Currently, it is not clear whether physical activity and sitting time change as a result of declining lung function, or whether low levels of physical activity and high levels of sitting time accelerate the decline in lung function. Given the previously described cyclical association between deconditioning and dyspnea, this association may be bi-directional. However, mechanistic studies are needed to understand how physical activity and sedentary time may affect outcomes such as lung function. For example, inflammation is associated with chronic lung disease, and is attenuated with participation in regular physical activity [45, 46], thus, it is possible that increasing physical activity and reducing sedentary time can directly affect respiratory disease physiology. With regard to healthcare use and quality of life, the role of functional autonomy, chronic comorbidities, medications, and disease control need to be assessed to better understand why physical activity and sitting time impact these outcomes.

## Conclusion

In conclusion, data from a nationally representative sample of adults with asthma, COPD, and lung function below LLN indicate that physical activity and sedentary time may be predictors of healthcare use and quality of life. This research may have implications related to the use of physical activity for improving health outcomes and quality of life among adults with obstructive lung disease.

## Additional files

Additional file 1: **Figure S1.** Crude associations of FEV1_%pred_ with Sitting Time and Physical Activity for adults with Asthma, COPD, and those below the LLN. Note: PA: Physical Activity; *p < 0.05, **p < 0.01, ***p < 0.001. (DOCX 23 kb)

Additional file 2: **Figure S2.** Crude associations of FEV_1_/FVC with Sitting Time and Physical Activity for adults with Asthma, COPD, and those below the LLN. Note: β values for the change in FEV_1_/FVC rather than the ratio (e.g. 70 to 70.2 rather than 0.70 to 0.702). PA: Physical Activity; *p < 0.05, **p < 0.01, ***p < 0.001. (DOCX 22 kb)

Additional file 3: **Figure S3.** Crude associations of Self-perceived health with Sitting Time and Physical Activity among adults with Asthma, COPD, and those below the LLN. Note: A higher OR indicates a higher odds of having “Poor” self-perceived health relative to the referent category. PA: Physical Activity; *p < 0.05, **p < 0.01, ***p < 0.001. (DOCX 23 kb)

Additional file 4: **Figure S4.** Crude associations of Self-perceived mental health with Sitting Time and Physical Activity among adults with Asthma, COPD, and those below the LLN. Note: A higher OR indicates a higher odds of having “Poor” self-perceived mental health relative to the referent category. PA: Physical Activity; *p < 0.05, **p < 0.01, ***p < 0.001. (DOCX 24 kb)

Additional file 5: **Figure S5.** Crude associations of Self-reported healthy aging with Sitting Time and Physical Activity among adults with Asthma, COPD, and those below the LLN. Note: A higher OR indicates a higher odds of having “Poor” self-reported healthy aging relative to the referent category. PA: Physical Activity; *p < 0.05, **p < 0.01, ***p < 0.001. (DOCX 23 kb)

## Abbreviations

CI: Confidence interval
CLSA: Canadian Longitudinal Study on Aging
COPD: Chronic obstructive pulmonary disease
FEV_1_: Forced expiratory volume in 1 seconds
FVC: Forced vital capacity
LLN: Lower limit of normal
PA: Physical activity
PASE: Physical Activity Scale for Elderly
