# Supplementary material for: Physical activity and sedentary time are related to clinically relevant health outcomes among adults with obstructive lung disease
Source: BMC Pulm Med. 2018 Jun 7;18:98. doi: 10.1186/s12890-018-0659-8 (PMC5992845; doi:10.1186/s12890-018-0659-8)
Supplement: Supplementary file 1 — Figure S1. Crude associations of FEV1%pred with Sitting Time and Physical Activity for adults with Asthma, COPD, and those below the LLN. Note: PA: Physical Activity; *p < 0.05, **p < 0.01, ***p < 0.001. (DOCX 23 kb) [file 12890_2018_659_MOESM1_ESM.docx]

Higher FEV_1%pred_

Lower FEV_1%pred_
