# Supplementary material for: Physical activity and sedentary time are related to clinically relevant health outcomes among adults with obstructive lung disease
Source: BMC Pulm Med. 2018 Jun 7;18:98. doi: 10.1186/s12890-018-0659-8 (PMC5992845; doi:10.1186/s12890-018-0659-8)
Supplement: Supplementary file 2 — Figure S2. Crude associations of FEV1/FVC with Sitting Time and Physical Activity for adults with Asthma, COPD, and those below the LLN. Note: β values for the change in FEV1/FVC rather than the ratio (e.g. 70 to 70.2 rather than 0.70 to 0.702). PA: Physical Activity; *p < 0.05, **p < 0.01, ***p < 0.001. (DOCX 22 kb) [file 12890_2018_659_MOESM2_ESM.docx]

Lower FEV_1_/FVC

Higher FEV_1_/FVC
