# Supplementary material for: Physical activity and sedentary time are related to clinically relevant health outcomes among adults with obstructive lung disease
Source: BMC Pulm Med. 2018 Jun 7;18:98. doi: 10.1186/s12890-018-0659-8 (PMC5992845; doi:10.1186/s12890-018-0659-8)
Supplement: Supplementary file 4 — Figure S4. Crude associations of Self-perceived mental health with Sitting Time and Physical Activity among adults with Asthma, COPD, and those below the LLN. Note: A higher OR indicates a higher odds of having “Poor” self-perceived mental health relative to the referent category. PA: Physical Activity; *p < 0.05, **p < 0.01, ***p < 0.001. (DOCX 24 kb) [file 12890_2018_659_MOESM4_ESM.docx]

Good Perceived Mental Health

Poor Perceived Mental Health
